# Impact of best response to ibrutinib plus Bendamustine and rituximab on PFS in MCL: a secondary analysis of SHINE

**DOI:** 10.1007/s00277-025-06569-7

**Published:** 2025-09-13

**Authors:** Yuko Mishima, Daigo Hashimoto, Michiko Ichii, Noriko Fukuhara, Toshiki Uchida, Koji Kato, Ai Omi, Yosuke Koroki, Kaname Shiga, Dai Maruyama

**Affiliations:** 1 Department of Hematology Oncology, Cancer Institute Hospital, Japanese Foundation for Cancer Research, Tokyo, Japan; 2 Department of Hematology, Hokkaido University Faculty of Medicine, Sapporo, Japan; 3 Department of Hematology and Oncology, Graduate School of Medicine, Osaka University, Osaka, Japan; 4 Department of Hematology, Tohoku University Hospital, Sendai, Japan; 5 Department of Hematology and Oncology, Japanese Red Cross Aichi Medical Center Nagoya Daini Hospital, Nagoya, Japan; 6 Department of Medicine and Biosystemic Science, Kyushu University Faculty of Medicine, Fukuoka, Japan; 7 Medical Affairs Division, Johnson & Johnson, Tokyo, Japan

**Keywords:** Bendamustine, Ibrutinib, Mantle cell lymphoma, Progression-free survival, Rituximab

## Abstract

**Abstract:**

Ibrutinib is a first-in-class oral inhibitor of Bruton’s tyrosine kinase, which was investigated for the first-line treatment of mantle cell lymphoma (MCL) in the randomized, double-blind, phase 3 SHINE study. In SHINE, ibrutinib plus bendamustine and rituximab (BR) demonstrated superior progression-free survival (PFS) versus placebo plus BR in patients aged ≥ 65 years with previously untreated stage II–IV MCL. In this secondary efficacy analysis of SHINE, we assessed the correlation between best response and PFS (*n* = 523; 70% male; median age 71.0 years). After a median follow-up of 94.5 months, patients achieving complete response (CR) had longer median PFS in the ibrutinib (97.8 months) and placebo (87.9 months) arms than those with partial response (PR; 27.6 and 16.7 months, respectively) or progressive/stable disease (2.9 and 3.4 months, respectively). In the multivariate logistic regression analysis, patients receiving ibrutinib plus BR were more likely to achieve CR than those receiving placebo plus BR (odds ratio 1.48; 95% confidence interval 1.00–2.22). In conclusion, prolonged long-term PFS was more likely in patients with MCL who achieved CR following treatment with either ibrutinib plus BR or placebo plus BR, while CR was more likely in patients who had received ibrutinib plus BR.

**Trial registration:**

EU Clinical Trials Register (EudraCT) identifier 2012-004056-11 (registered 15 January 2013) WHO Universal Trial Number U1111-1137-0389.

**Supplementary Information:**

The online version contains supplementary material available at 10.1007/s00277-025-06569-7.

## Introduction

Mantle cell lymphoma (MCL), a subtype of non-Hodgkin lymphoma [1], is often characterized by an aggressive disease course, necessitating systemic treatment [2]. However, patients with MCL tend to be aged > 65 years and are thus considered to be unsuitable candidates for high-dose chemotherapy and hematopoietic stem cell transplantation [3].

Ibrutinib is a first-in-class oral inhibitor of Bruton’s tyrosine kinase [4]. The SHINE study (NCT01776840) was a randomized, double-blind, phase 3 trial of patients aged ≥ 65 years with previously untreated stage II–IV MCL [5]. The primary analysis of SHINE showed that the combination of ibrutinib plus bendamustine and rituximab (BR), followed by rituximab maintenance therapy, significantly prolonged progression-free survival (PFS; primary endpoint) compared with placebo plus BR; overall survival (OS) was similar between the treatment groups [5]. Although ibrutinib plus BR favored PFS in most of the pre-specified subgroup analyses [5], it was unclear which patients achieved the greatest PFS benefit. In a previous pooled analysis of data from three studies of second-line ibrutinib in relapsed/refractory MCL (median follow-up 9.7 years), patients with a best response of complete response (CR) had prolonged PFS versus those with partial response (PR) [6], indicating that good treatment response may result in better long-term PFS than incomplete/poor response.

A post-hoc secondary analysis of SHINE was conducted, focusing on evaluating the efficacy of ibrutinib plus BR in patients with MCL according to treatment response.

## Methods

The design of the SHINE study, conducted across 183 sites in North America, South America, Europe, Asia, and Oceania, has been previously described in detail [5]. Briefly, patients aged ≥ 65 years with previously untreated stage II–IV MCL were randomized (stratified by simplified Mantle Cell Lymphoma International Prognostic Index [sMIPI] score category) to receive either ibrutinib 560 mg orally once daily or placebo, in combination with six cycles of bendamustine (90 mg/m^2^ body surface area [BSA] on days 1 and 2 of each 28-day cycle) and rituximab (375 mg/m^2^ BSA on day 1 of each cycle). Patients with CR or PR after six cycles of treatment continued to receive rituximab maintenance therapy every 8 weeks for up to 12 additional doses and treatment was administered until disease progression or unacceptable toxic effects occurred.

In this secondary analysis, outcomes were assessed by best response subgroups, namely in patients who achieved a CR, who achieved a PR, and those with stable disease (SD) or progressive disease (PD). Best response was evaluated using computed tomography (CT) scans of the neck, chest, abdomen, pelvis, and any other location where disease was present or by magnetic resonance imaging (MRI) scans if disease sites could not be adequately determined by CT. Radiologic imaging assessments were carried out at screening, then (after the initiation of ibrutinib or placebo) every 12 weeks during the first 12 months, and then every 16 weeks until disease progression occurred. To confirm CR, a positron-emission tomographic (PET) scan was conducted. Patients with bone marrow involvement at baseline were required to undergo a repeat bone marrow evaluation at the time of determining CR, while an endoscopy was required to confirm a CR in those with known gastrointestinal involvement at baseline. Disease progression was assessed according to the 2007 Revised Response Criteria for Malignant Lymphoma [7].

PFS, OS, and time to next treatment (TTNT) were estimated using the Kaplan–Meier method in patients who achieved CR, PR, or SD or PD. For PFS, the hazard ratio (HR) and associated 95% confidence interval (CI) were calculated using a stratified Cox proportional-hazards model. For OS and TTNT, the stratified log-rank test was used for between-treatment arm comparisons. Response rates were compared using the Cochran–Mantel–Haenszel test.

Univariate and multivariate analyses were conducted, using factors with known negative impact on outcomes, to identify predictors of achieving CR versus PR. *TP53* was not included in the multivariate analysis because it is not included in either the sMIPI or revised Mantle Cell Lymphoma International Prognostic Index (MIPI). Eastern Cooperative Oncology Group performance status (ECOG PS) was also not included in the multivariate analysis because SHINE only included patients with ECOG PS of 0 or 1. Finally, since SHINE included patients aged ≥ 65 years, the threshold age was set at 75 years based on the univariate analysis.

## Results

Of the 589 patients screened for SHINE, 523 were randomized to receive either ibrutinib plus BR (*n* = 261) or placebo plus BR (*n* = 262). Among those randomized to ibrutinib plus BR, 171 patients (65.5%) achieved CR, 63 (24.1%) achieved PR, and 27 (10.3%) had SD or PD (Supplemental Fig. 1). Among those randomized to placebo plus BR, 151 patients (57.6%) achieved CR, 81 (30.9%) achieved PR, and 30 (11.5%) had SD or PD.

**Fig. 1:**
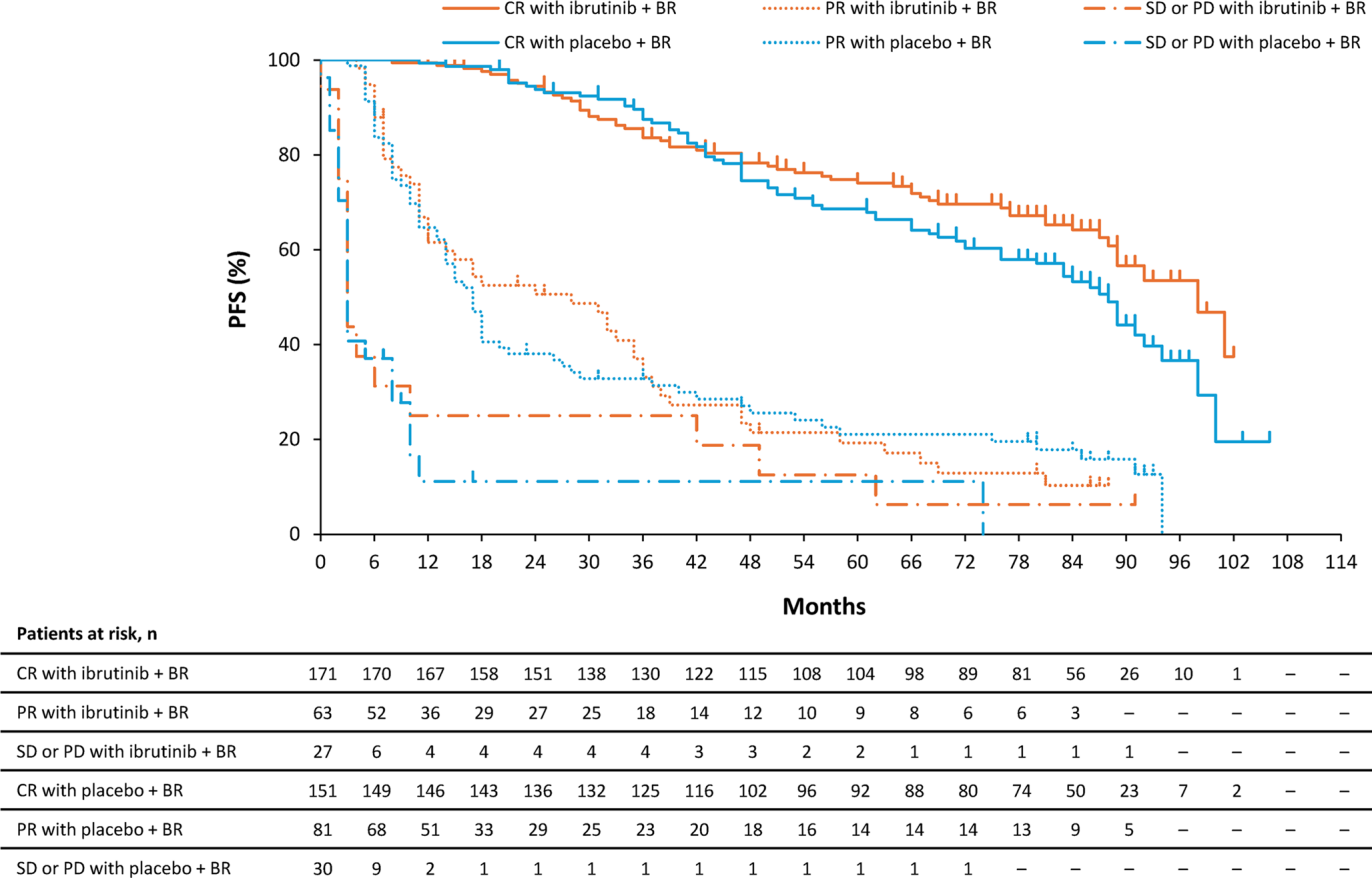
Progression-free survival by best response. BR, bendamustine and rituximab; CR, complete response; PD, progressive disease; PFS, progression-free survival; PR, partial response; SD, stable disease

The baseline characteristics of the patients in SHINE were generally well balanced across both treatment arms in the original analysis [5]. Although there was some variation in patient baseline characteristics across the different response categories in this secondary analysis, they remained balanced between the treatment arms (Table 1). There were notable differences in the median duration of treatment between patients who achieved CR or PR, and those who had SD or PD, as well as between the ibrutinib plus BR and placebo plus BR arms (Table 2). Relative dose intensity was consistently high across both treatment arms and across all response categories.

**Table 1:** Baseline demographic and clinical characteristics of the included patients by best response

	Ibrutinib plus BR (*N* = 261)	Placebo plus BR (*N* = 262)	Ibrutinib plus BR, by treatment response			Placebo plus BR, by treatment response		
			CR (*n* = 171)	PR (*n* = 63)	SD or PD (*n* = 27)	CR (*n* = 151)	PR (*n* = 81)	SD or PD (*n* = 30)
Age, years, median (range)	71.0 (65–86)	71.0 (65–87)	71.0 (65–86)	72.0 (65–84)	72.0 (65–85)	71.0 (65–87)	72.0 (65–86)	71.0 (65–82)
Age category, n (%)								
³75 years	74 (28.4)	82 (31.3)	48 (28.1)	17 (27.0)	9 (33.3)	41 (27.2)	31 (38.3)	10 (33.3)
Sex, n (%)								
Male	178 (68.2)	186 (71.0)	116 (67.8)	49 (77.8)	13 (48.1)	98 (64.9)	62 (76.5)	26 (86.7)
ECOG PS, n (%)								
0	134 (51.3)	141 (53.8)	100 (58.5)	26 (41.3)	8 (29.6)	95 (62.9)	34 (42.0)	12 (40.0)
1	127 (48.7)	118 (45.0)	71 (41.5)	37 (58.7)	19 (70.4)	56 (37.1)	45 (55.6)	17 (56.7)
≥ 2	0	3 (1.1)	0	0	0	0	2 (2.5)	1 (3.3)
sMIPI, n (%)								
Low risk	44 (16.9)	46 (17.6)	36 (21.1)	5 (7.9)	3 (11.1)	37 (24.5)	8 (9.9)	1 (3.3)
Intermediate risk	124 (47.5)	129 (49.2)	90 (52.6)	26 (41.3)	8 (29.6)	72 (47.7)	49 (60.5)	8 (26.7)
High risk	93 (35.6)	87 (33.2)	45 (26.3)	32 (50.8)	16 (59.3)	42 (27.8)	24 (29.6)	21 (70.0)
Blastoid or pleomorphic histology, n (%)	19 (7.3)	26 (9.9)	9 (5.3)	8 (12.7)	2 (7.4)	9 (6.0)	12 (14.8)	5 (16.7)
Blastoid	14 (5.4)	24 (9.2)	6 (3.5)	6 (9.5)	2 (7.4)	9 (6.0)	10 (12.3)	5 (16.7)
Pleomorphic	5 (1.9)	2 (0.8)	3 (1.8)	2 (3.2)	0	0	2 (2.5)	0
Bone marrow involvement, n (%)	198 (75.9)	200 (76.3)	127 (74.3)	50 (79.4)	21 (77.8)	109 (72.2)	66 (81.5)	25 (83.3)
Extranodal, n (%)	234 (89.7)	226 (86.3)	150 (87.7)	60 (95.2)	24 (88.9)	121 (80.1)	77 (95.1)	28 (93.3)
Tumor bulk, n (%)	*n* = 260	*n* = 261	*n* = 171	*n* = 62	*n* = 27	*n* = 151	*n* = 81	*n* = 29
≥ 5 cm	95 (36.5)	98 (37.5)	63 (36.8)	26 (41.9)	6 (22.2)	48 (31.8)	36 (44.4)	14 (48.3)
*TP53*, n (%)	*n* = 140	*n* = 129	*n* = 96	*n* = 35	*n* = 9	*n* = 70	*n* = 48	*n* = 11
Mutated	26 (18.6)	24 (18.6)	11 (11.5)	12 (34.3)	3 (33.3)	7 (10.0)	14 (29.2)	3 (27.3)
LDH, n (%)	*n* = 260	*n* = 259	*n* = 170	*n* = 63	*n* = 27	*n* = 150	*n* = 81	*n* = 28
≥ULN	86 (33.1)	79 (30.5)	50 (29.4)	24 (38.1)	12 (44.4)	35 (23.3)	27 (33.3)	17 (60.7)
WBC count, n (%)	*n* = 261	*n* = 260	*n* = 171	*n* = 63	*n* = 27	*n* = 151	*n* = 81	*n* = 28
≥ 15,000 cells/µL	45 (17.2)	55 (21.2)	22 (12.9)	12 (19.0)	11 (40.7)	30 (19.9)	11 (13.6)	14 (50.0)
Albumin, n (%)	*n* = 257	*n* = 256	*n* = 168	*n* = 62	*n* = 27	*n* = 150	*n* = 80	*n* = 26
≥ 35 g/L	202 (78.6)	196 (76.6)	136 (81.0)	45 (72.6)	21 (77.8)	125 (83.3)	54 (67.5)	17 (65.4)

BR, bendamustine and rituximab; CR, complete response; ECOG PS, Eastern Cooperative Oncology Group performance status; LDH, lactate dehydrogenase; PD, progressive disease; PR, partial response; SD, stable disease; sMIPI, simplified Mantle Cell Lymphoma International Prognostic Index; *TP53*, tumor protein P53; ULN, upper limit of normal; WBC, white blood cell

**Table 2:** Drug exposure by best response

	Ibrutinib plus BR			Placebo plus BR		
	CR (*n* = 171)	PR (*n* = 63)	SD or PD (*n* = 25)	CR (*n* = 151)	PR (*n* = 81)	SD or PD (*n* = 28)
Total treatment duration, months, median (range)	43.17 (0.7–106.2)	9.69 (0.9–96.9)	1.51 (0.2–3.2)	55.92 (1.7–100.1)	13.86 (1.3–97.1)	2.00 (0.0–12.0)
Dose per administration, mg/day, median (range)	532.06 (110.5–566.8)	533.83 (191.6–560.0)	560.00 (135.3–560.0)	549.23 (145.3–560.0)	551.90 (226.7–560.8)	560.00 (60.0–560.0)
RDI, %, median (range)	95.01 (19.7–101.2)	95.33 (34.2–100.0)	100.00 (24.2–100.0)	98.08 (25.9–100.0)	98.55 (40.5–100.1)	100.00 (10.7–100.0)
RDI category, n (%)						
< 70%	22 (12.9)	7 (11.1)	4 (16.0)	8 (5.3)	2 (2.5)	5 (17.9)
≥ 70% to < 90%	32 (18.7)	11 (17.5)	5 (20.0)	15 (9.9)	10 (12.3)	3 (10.7)
≥ 90%	117 (68.4)	45 (71.4)	16 (64.0)	128 (84.8)	69 (85.2)	20 (71.4)

BR, bendamustine and rituximab; CR, complete response; RDI, relative dose intensity; PD, progressive disease; PR, partial response; SD, stable disease

After a median follow-up of 94.5 months (9.8 months longer than the median follow-up in the primary efficacy analysis of SHINE [5]) in the ibrutinib plus BR arm, the median (95% CI) PFS was 97.8 months (89.5 months–not reached [NR]), 27.6 months (12.0–34.8), and 2.9 months (2.4–10.3) among patients who achieved CR, PR and with SD or PD, respectively (Fig. 1). In the placebo plus BR arm, the median (95% CI) PFS was 87.9 months (76.4–91.6), 16.7 months (13.8–19.8), and 3.4 months (2.2–8.3) among those who achieved CR, PR, and with SD or PD, respectively. TTNT (Supplementary Fig. 2) and OS (Supplementary Fig. 3) showed similar trends. The CR rate tended to be higher in the ibrutinib plus BR arm versus the placebo plus BR arm after Week 36 (Supplementary Fig. 4).

The univariate analysis (Supplementary Table 1) showed that the likelihood of achieving CR was higher among patients with albumin levels ≥ 35 g/L at baseline (vs. < 35 g/L), and lower among patients with an ECOG PS of ≥ 1 (vs. 0), and with blastoid or pleomorphic histology (vs. other histology), sMIPI intermediate risk (vs. low risk) or high risk (vs. low risk), and a *TP53* mutation (vs. no mutation).

In the multivariate analysis, the likelihood of achieving CR was higher among patients treated with ibrutinib plus BR versus placebo plus BR (Table 3). Other factors that influenced the likelihood of achieving CR were age ≥ 75 years (vs. < 75 years), lactate dehydrogenase (LDH) levels ≥ the upper limit of normal (ULN; vs. < ULN), bone marrow involvement (vs. no bone marrow involvement), and white blood cell (WBC) count ≥ 15,000 cells/µL (vs. < 15,000 cells/µL; Table 3).

**Table 3:** Multivariate logistic regression model predicting the best response

Parameter	CR (*n* = 322)	PR (*n* = 144)	OR^a^ (95% CI)
Treatment arm, n (%)			
Ibrutinib plus BR	170 (52.8)	63 (43.8)	1.48 (1.00–2.22)
Placebo plus BR	150 (46.6)	81 (56.3)	Ref
Age, n (%)			
< 75 years	231 (71.7)	96 (66.7)	Ref
≥ 75 years	89 (27.6)	48 (33.3)	0.80 (0.52–1.22)
LDH, n (%)			
≥ULN	85 (26.4	51 (35.4)	0.66 (0.42–1.01)
< ULN	235 (73.0)	93 (64.6)	Ref
Bone marrow involvement, n (%)			
Yes	234 (72.7)	116 (80.6)	0.68 (0.41–1.11)
No	86 (26.7)	28 (19.4)	Ref
WBC count, n (%)			
≥ 15,000 cells/µL	51 (15.8)	23 (16.0)	1.24 (0.71–2.17)
< 15,000 cells/µL	269 (83.5)	121 (84.0)	Ref

^a^For CR vs. PRBR, bendamustine and rituximab; CI, confidence interval; CR, complete response; LDH, lactate dehydrogenase; OR, odds ratio; PR, partial response; Ref, reference; ULN, upper limit of normal; WBC, white blood cell

## Discussion

The results of this post-hoc analysis suggest that CR is associated with better long-term disease control and improved survival outcomes in MCL. Several prognostic factors for MCL have been previously identified. The MIPI was developed in 2008 using data from 455 patients with advanced-stage MCL, and includes four independent prognostic factors (age, ECOG PS, LDH level, and WBC count) [8]. Subsequently, a retrospective analysis of 501 Japanese patients with MCL who received rituximab-containing immunotherapy showed that, in addition to the MIPI prognostic factors, low serum albumin and bone marrow involvement were also associated with poorer outcomes [9]. In the current analysis, predictors of achieving CR were evaluated through logistic regression analyses. Based on the results of the multivariate analysis, after adjusting for MIPI-related covariates, treatment with ibrutinib plus BR remained a potential predictive factor of achieving CR.

The results of this secondary efficacy analysis of SHINE highlight that prolonged PFS was more likely in patients with MCL who achieved CR following treatment with either ibrutinib plus BR or placebo plus BR. The relationship between best response and PFS has been well-documented in non-Hodgkin lymphoma; a meta-analysis of 73 clinical trials showed a strong correlation between objective response rate and median PFS in patients with non-Hodgkin lymphoma, including MCL [10]. This is in line with the findings of the present analysis, which showed that achieving CR correlated with prolonged PFS. Patients who achieve CR with manageable adverse events are recommended to continue treatment with ibrutinib, and the result of other BTK inhibitor’s study also suggests that combining a BTK inhibitor with BR may offer additional benefits [11]. Those benefits included significantly improved PFS, higher overall response and CR rates, as well as a similar adverse event profile, compared with the BR-only regimen.

The present analysis had some limitations SHINE included only older patients but excluded patients with significant comorbidities or poor ECOG PS. This may limit the generalizability of the findings to other populations. In addition, the controlled clinical trial setting may not fully replicate real-world conditions. Finally, determining the timepoint of the best response was not aligned for all patients, so the analyses of survival function may have been subject to an immortal time bias.

In conclusion, this post-hoc secondary efficacy analysis of the SHINE study showed that prolonged PFS was more likely in patients with MCL who achieved CR, and that CR was more likely with ibrutinib plus BR than placebo plus BR.

## Supplementary Information

Below is the link to the electronic supplementary material.

Supplementary Material 1 (DOCX. 372 KB)

## Data Availability

The datasets used and/or analyzed during the current study are available on reasonable request through the Yale Open Data Access (YODA) Project site at http://yoda.yale.edu.

## References

[1] Li JY, Gaillard F, Moreau A et al (1999) Detection of translocation t(11;14)(q13;q32) in mantle cell lymphoma by fluorescence in situ hybridization. Am J Pathol 154:1449–1452. 10.1016/s0002-9440(10)65399-0 10329598 10.1016/S0002-9440(10)65399-0 PMC1866594

[2] Jain P, Wang M (2019) Mantle cell lymphoma: 2019 update on the diagnosis, pathogenesis, prognostication, and management. Am J Hematol 94:710–725. 10.1002/ajh.25487 30963600 10.1002/ajh.25487

[3] Robak T, Smolewski P, Robak P, Dreyling M (2019) Mantle cell lymphoma: therapeutic options in transplant-ineligible patients. Leuk Lymphoma 60:2622–2634. 10.1080/10428194.2019.1605511 31018735 10.1080/10428194.2019.1605511

[4] Kim ES, Dhillon S (2015) Ibrutinib: a review of its use in patients with mantle cell lymphoma or chronic lymphocytic leukaemia. Drugs 75:769–776. 10.1007/s40265-015-0380-3 25802231 10.1007/s40265-015-0380-3

[5] Wang ML, Jurczak W, Jerkeman M et al (2022) Ibrutinib plus Bendamustine and rituximab in untreated mantle-cell lymphoma. N Engl J Med 386:2482–2494. 10.1056/NEJMoa2201817 35657079 10.1056/NEJMoa2201817

[6] Dreyling M, Goy A, Hess G et al (2022) Long-term outcomes with ibrutinib treatment for patients with relapsed/refractory mantle cell lymphoma: a pooled analysis of 3 clinical trials with nearly 10 years of follow-up. Hemasphere 6:e712. 10.1097/HS9.0000000000000712 35441128 10.1097/HS9.0000000000000712 PMC9010121

[7] Cheson BD, Pfistner B, Juweid ME et al (2007) Revised response criteria for malignant lymphoma. J Clin Oncol 25:579–586. 10.1200/jco.2006.09.2403 17242396 10.1200/JCO.2006.09.2403

[8] Hoster E, Dreyling M, Klapper W et al (2008) A new prognostic index (MIPI) for patients with advanced-stage mantle cell lymphoma. Blood 111:558–565. 10.1182/blood-2007-06-095331 17962512 10.1182/blood-2007-06-095331

[9] Chihara D, Asano N, Ohmachi K et al (2015) Prognostic model for mantle cell lymphoma in the rituximab era: a nationwide study in Japan. Br J Haematol 170:657–668. 10.1111/bjh.13486 25953436 10.1111/bjh.13486

[10] Mangal N, Salem AH, Li M, Menon R, Freise KJ (2018) Relationship between response rates and median progression-free survival in non-Hodgkin’s lymphoma: a meta-analysis of published clinical trials. Hematol Oncol 36:37–43. 10.1002/hon.2463 28707346 10.1002/hon.2463

[11] Wang M, Salek D, Belada D et al (2025) Acalabrutinib plus bendamustine-rituximab in untreated mantle cell lymphoma. J Clin Oncol 43:2276–2284. 10.1200/JCO-25-00690 40311141 10.1200/JCO-25-00690 PMC12225732

