# Supplementary material for: Impact of best response to ibrutinib plus Bendamustine and rituximab on PFS in MCL: a secondary analysis of SHINE
Source: Ann Hematol. 2025 Sep 13;104(9):4605–10. doi: 10.1007/s00277-025-06569-7 (PMC12552230; doi:10.1007/s00277-025-06569-7)
Supplement: Supplementary file 1 — Supplementary Material 1 (DOCX. 372 KB) [file 277_2025_6569_MOESM1_ESM.pdf]

Impact of best response to ibrutinib plus bendamustine and rituximab on PFS in MCL: a secondary analysis of SHINE

Annals of Hematology

Authors: Yuko Mishima,[1] Daigo Hashimoto,[2] Michiko Ichii,[3] Noriko Fukuhara,[4] Toshiki Uchida,[5] Koji Kato,[6] Ai Omi,[7] Yosuke Koroki,[7] Kaname Shiga,[7] Dai Maruyama[1]

Corresponding author: Yosuke Koroki, Department of Medical Affairs, Johnson & Johnson, 3-5-2 Nishi-kanda, Chiyoda-ku, Tokyo 101-0065, Japan. Tel: +81-80-8130-5336; E-mail: ykoroki@its.jnj.com

Supplementary information

Supplementary Table 1 Univariate logistic regression model predicting the best response

**Parameter**	**CR (n=322)**	**PR (n=144)**	**OR[a] (95% CI)**
Treatment arm, n (%)			
Ibrutinib plus BR	171 (53.1)	63 (43.8)	1.46 (0.98–2.16)
Placebo plus BR	151 (46.9)	81 (56.3)	Ref
Age category, n (%)			
<70 years	132 (41.0)	51 (35.4)	Ref
≥70 years	190 (59.0)	93 (64.6)	0.79 (0.53–1.19)
<75 years	233 (72.4)	96 (66.7)	Ref
≥75 years	89 (27.6)	48 (33.3)	0.76 (0.50–1.17)
LDH, n (%)	n=320		
≥ULN	85 (26.6)	51 (35.4)	0.66 (0.43–1.01)
<ULN	235 (73.4)	93 (64.6)	Ref
Bone marrow involvement, n (%)			
Yes	236 (73.3)	116 (80.6)	0.66 (0.41–1.07)
No	86 (26.7)	28 (19.4)	Ref
WBC count, n (%)			
≥15,000 cells/µL	52 (16.1)	23 (16.0)	1.01 (0.59–1.73)
<15,000 cells/µL	270 (83.9)	121 (84.0)	Ref
Albumin, n (%)	n=318	n=142	
<35 g/L	57 (17.9)	43 (30.3)	Ref
≥35 g/L	261 (82.1)	99 (69.7)	1.99 (1.26–3.15)
ECOG PS, n (%)			
0	195 (60.6)	60 (41.7)	Ref
≥1	127 (39.4)	84 (58.3)	0.47 (0.31–0.69)
Blastoid/pleomorphic histology, n (%)	n=272	n=131	
Yes	18 (6.6)	20 (15.3)	0.39 (0.20–0.77)
No	254 (93.4)	111 (84.7)	Ref
sMIPI, n (%)			
Low risk	73 (22.7)	13 (9.0)	Ref
Intermediate risk	162 (50.3)	75 (52.1)	0.38 (0.20–0.74)
High risk	87 (27.0)	56 (38.9)	0.28 (0.14–0.55)
TP53, n (%)	n=166	n=83	
Mutated	18 (10.8)	26 (31.3)	0.27 (0.14–0.52)
Unmutated	148 (89.2)	57 (68.7)	Ref

[a]For CR vs PR

BR, bendamustine and rituximab; CI, confidence interval; CR, complete response; ECOG PS, Eastern Cooperative Oncology Group performance status; LDH, lactate dehydrogenase; OR, odds ratio; PR, partial response; Ref, reference; sMIPI, simplified Mantle Cell Lymphoma International Prognostic Index; *TP53*, tumor protein P53; ULN, upper limit of normal; WBC, white blood cell.

Supplementary Fig. 1 Patient disposition

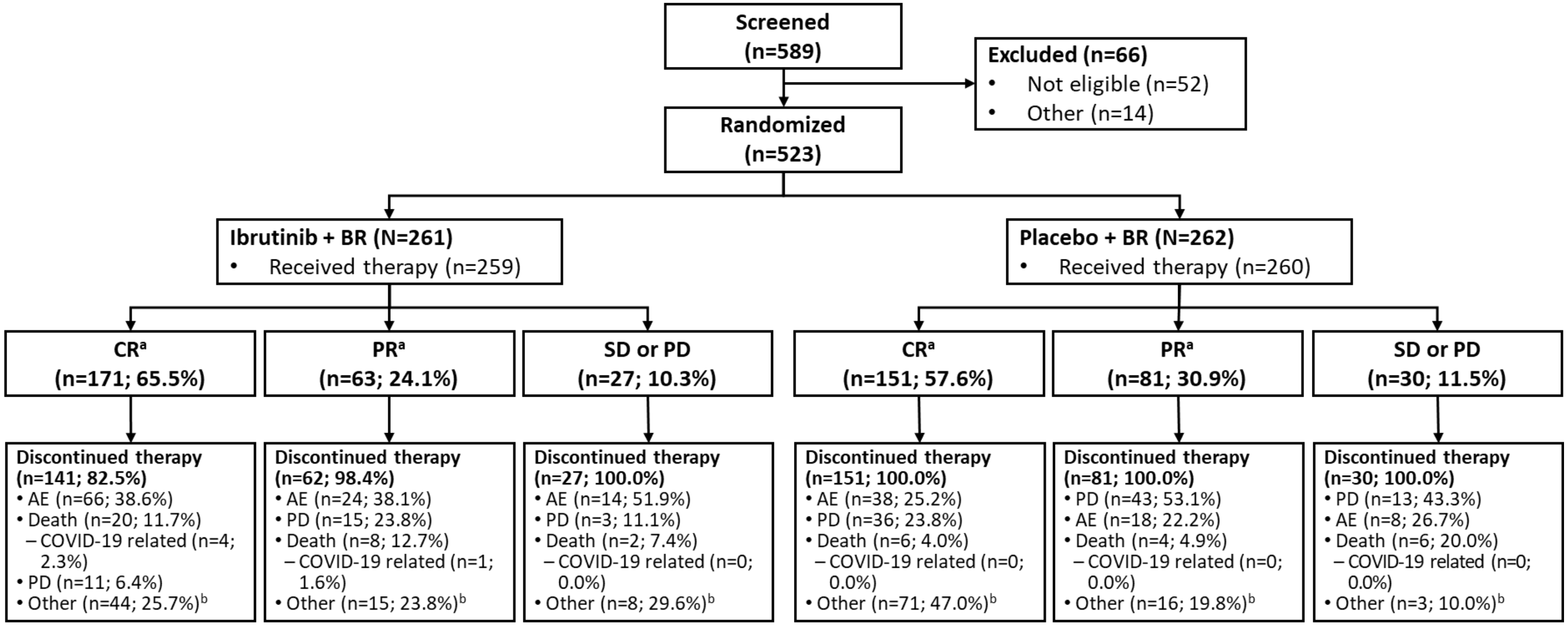

[a]There was no statistically significant difference in CR rate and ORR between treatment arms.

[b]Other reasons for discontinued therapy included withdrawal of consent, investigator or sponsor decision, and lost to follow up.

AE, adverse event; BR, bendamustine and rituximab; CR, complete response; ORR, objective response rate; PD, progressive disease; PR, partial response; SD, stable disease.

Supplementary Fig. 2 Time to next treatment by best response

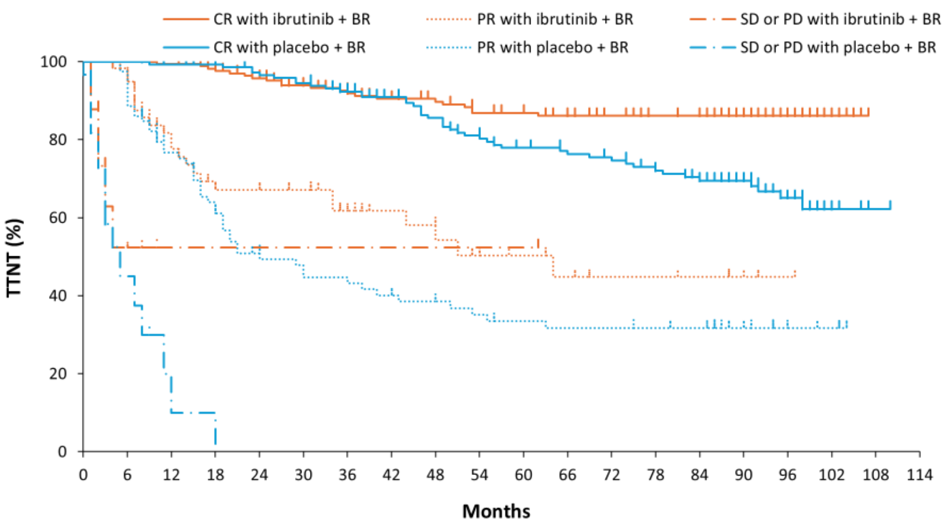

Patients at risk, n																				
CR with ibrutinib + BR	171	171	169	161	154	145	134	129	124	118	115	111	105	98	94	84	47	11	–	–
PR with ibrutinib + BR	63	56	39	30	29	28	20	17	15	12	10	8	6	6	5	3	1	–	–	–
SD or PD with ibrutinib + BR	27	4	1	1	1	1	1	1	1	1	1	–	–	–	–	–	–	–	–	–
CR with placebo + BR	151	151	147	144	139	136	128	121	113	104	98	95	91	85	79	57	34	6	1	–
PR with placebo + BR	81	74	55	44	32	31	29	25	24	21	19	18	18	17	16	9	4	3	–	–
SD or PD with placebo + BR	30	6	1	1	–	–	–	–	–	–	–	–	–	–	–	–	–	–	–	–

BR, bendamustine and rituximab; CR, complete response; PD, progressive disease; PR, partial response; SD, stable disease; TTNT, time to next treatment.

Supplementary Fig. 3 Overall survival by best response

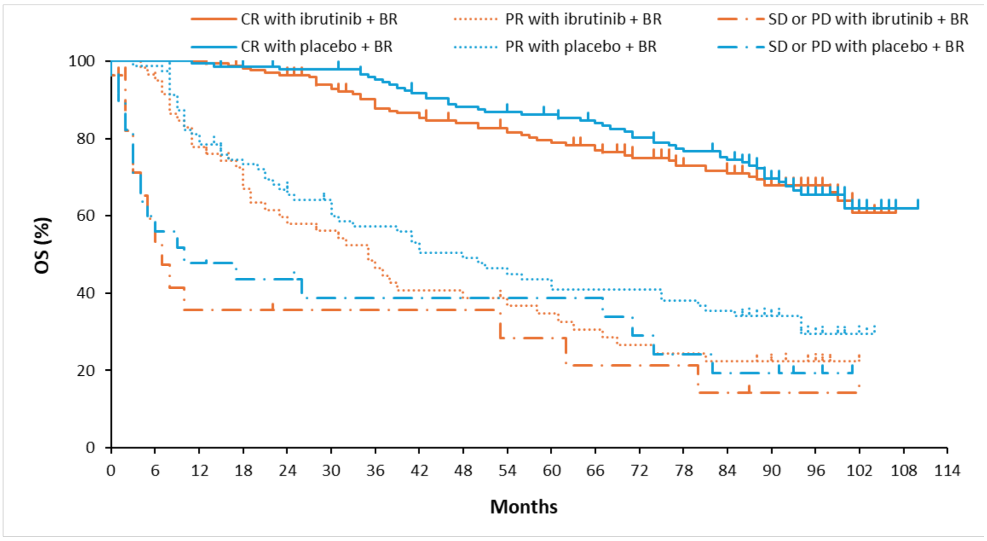

Patients at risk, n																				
CR with ibrutinib + BR	171	171	170	164	160	152	142	137	133	129	124	120	112	105	100	89	50	11	–	–
PR with ibrutinib + BR	63	58	45	38	32	30	24	21	20	19	17	15	13	12	11	9	6	1	–	–
SD or PD with ibrutinib + BR	27	10	6	6	5	5	5	5	5	4	4	3	3	3	2	1	1	1	–	–
CR with placebo + BR	151	151	148	145	143	143	138	131	126	123	121	116	111	106	102	78	45	11	1	–
PR with placebo + BR	81	79	63	57	50	46	42	38	37	34	30	30	30	28	26	17	10	4	–	–
SD or PD with placebo + BR	30	14	12	10	10	8	8	8	8	8	8	8	6	5	4	4	2	–	–	–

BR, bendamustine and rituximab; CR, complete response; OS, overall survival; PD, progressive disease; PR, partial response; SD, stable disease.

Supplementary Fig. 4 Objective response rate with (A) ibrutinib plus bendamustine and rituximab (B) and placebo plus bendamustine and rituximab by duration of follow-up

A

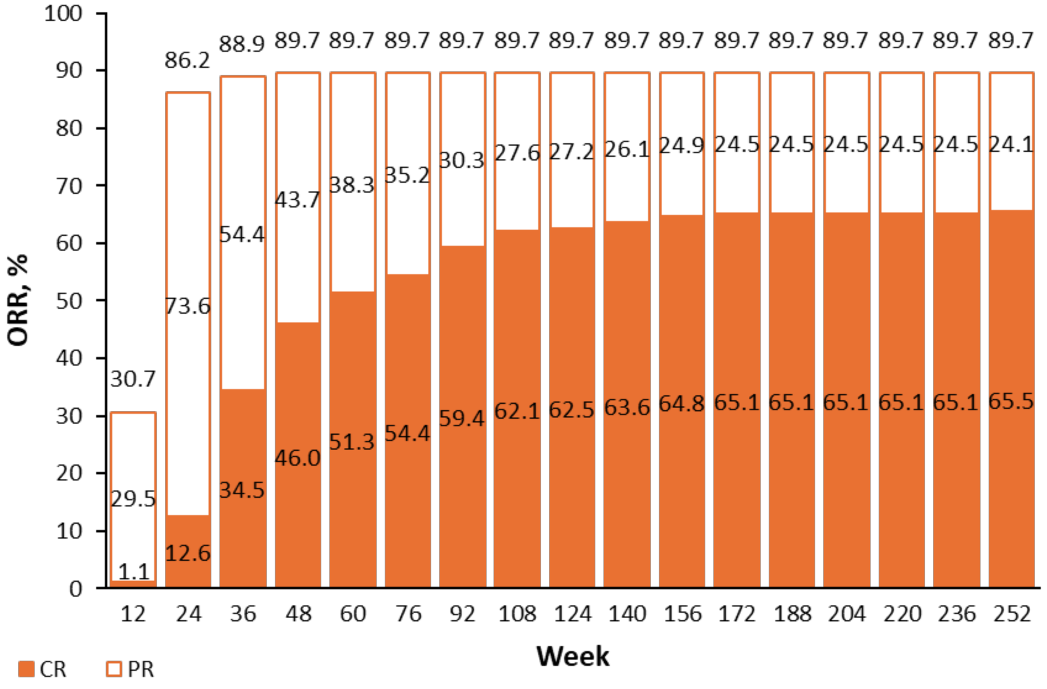

B

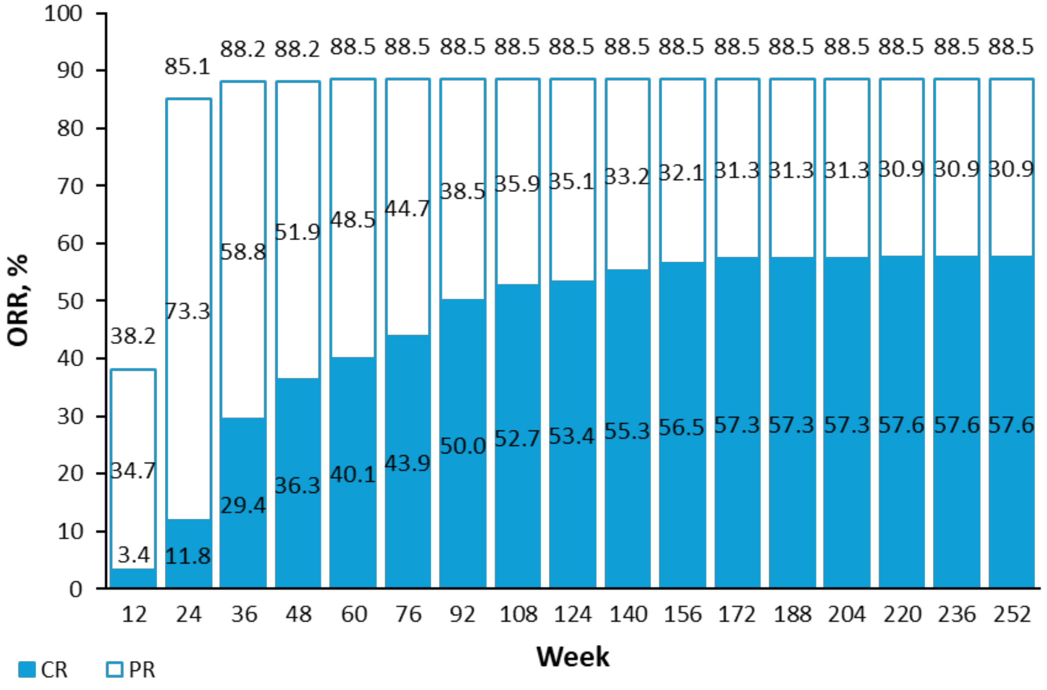

CR, complete response; ORR, objective response rate; PR, partial response.
